# Simulation of Different Truncated p16^INK4a^ Forms and *In Silico* Study of Interaction with Cdk4

**DOI:** 10.4137/cin.s878

**Published:** 2008-12-03

**Authors:** Najmeh Fahham, Mohammad Hossein Ghahremani, Soroush Sardari, Behrouz Vaziri, Seyed Nasser Ostad

**Affiliations:** 1 Department of Medical Biotechnology, Biotechnology Research Center, Pasteur Institute, Tehran, Iran 13164; 2 Department of Pharmacology–Toxicology, Faculty of Pharmacy, Tehran University of Medical Sciences, Tehran, Iran

**Keywords:** p16^INK4a^, Cdk4, protein-protein interaction, docking

## Abstract

Protein-protein interactions studies can greatly increase the amount of structural and functional information pertaining to biologically active molecules and processes. The information obtained from such studies can lead to design and application of new modification in order to obtain a desired bioactivity. Many application packages and servers performing docking, such as HEX, DOT, AUTODOCK, and ZDOCK are now available for predicting the lowest free energy state of a protein complex. In this study, we have focused on cyclin-dependent kinase 4 (Cdk4), a key molecule in the regulation of cell cycle progression at the G_1_-S phase restriction point and p16^INK4a^, a tumor suppressor which inhibits Cdk4 activity. Truncated structures were created to find the more critical regions of p16 for interaction. The tertiary structures were determined by ProSAL, GENO3D Web Server. We evaluated their interactions with Cdk4 using two docking systems, HEX 4.5 and DOT 1. Calculations were performed on a high-speed computer. Minimizations and visualizations were carried out by PdbViewer 3.7. Considering shape and shape/electrostatic total energy, structures containing ANK II, III and IV motifs that lack the N-terminal region of the full length p16 molecule showed the best fit complexes among the p16 truncated forms. The free energies were compatible with that of p16 full length original form, the full length. It seems that the N-terminal of the molecule is not crucial for the interaction since the truncated structure containing only this region did not show a good total energy.

## Introduction

Protein-protein interactions involve in a variety of cellular functions including protein localization, gene regulation, and signal transduction. Recent developments in the proteomics studies such as mass spectroscopy, yeast two-hybrid assays and display cloning reveal a considerable number of interacting proteins, among which a small fraction of the potential complexes will be amenable to direct experimental analysis. The computational prediction of protein-protein interactions can therefore increase the amount of structural protein interaction knowledge.

Progression through the different phases of the cell cycle is controlled at several checkpoints, specifically G_1_-S transition state (Villacanas et al. 2002). Genetic analysis has revealed that certain proteins involved in this phase of the cell cycle have most often altered in human tumors. In particular, the cyclin-dependent kinase (Cdk)-cyclin D/INK4/retinoblastoma (pRb) E2F cascade has been found to be altered in more than 80% of human neoplasias (Ortega et al. 2002). This process begins with the activation of the Cdk4/6 upon binding to cyclin D with the subsequent phosphorylation of pRb and release of the E2F transcription factor. The p16^INK4a^ tumor suppressor gene, located at the chromosomal 9p21 region, is a specific Cdk4/6 inhibitor (Kwong et al. 2004). Since it was first reported in 1994, p16^INK4a^ has been considered as one of the most altered genes in a wide variety of malignant human tumors (>70 different types). It has been inactivated by several molecular mechanisms including homozygous deletions, point mutations and hyper methylation in CpG islands (Xie et al. 2005). These inactivating mutations severely affect the stability of the tertiary structure of p16^INK4a^ and its function (Serrano, 1997).

**Figure 1:**
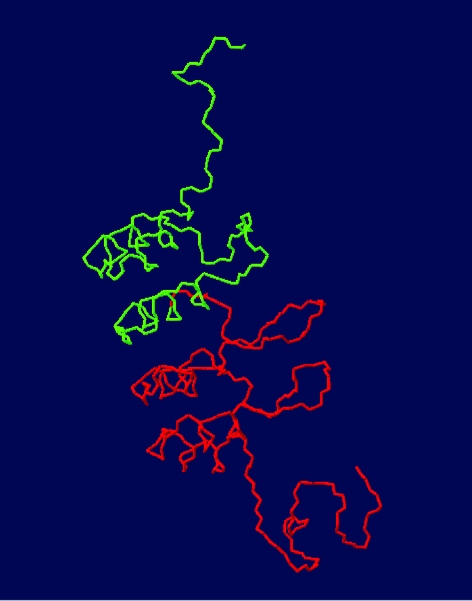
Solution NMR structure of tumor suppressor p16^INK4a^, minimized mean structure, Protein Data Bank, entry 2A5E, represented by PdbViewer 3.7 (2001). The two N, C-terminal halves have been represented in green and red, respectively.

**Figure 2:**
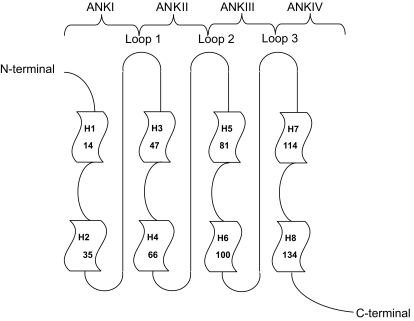
Topology diagram of the p16 structure. Each ankyrin repeat exhibits a helix-turn-helix structure. The helices are designated as 1, 2, 3, etc. The four H-T-H motifs are connected by three loops in beta and gamma turn structure (Byeon, Li et al. 1998).

p16 is comprised mainly of four ankyrin repeats, which are believed to be involved in protein-protein interactions. Figure 1 show the solution structure and Figure 2 presents the topology diagram of p16^INK4a^. The deletion of C-terminal portion after codon 135 has been shown to have no effect on the activity of p16 *in vitro*. On the other hand, according to random mutagenesis p16 gene studies, there is a large contact surface between p16 and Cdk4, while many amino acids throughout the four ankyrin repeats are important for the interaction (Yang et al. 1996). Although based on the molecular dynamic studies, changes that slightly affect the binding of p16 to Cdk4 can disrupt the normal function of p16, some interactions between the receptor and the inhibitor are functionally redundant (Villacanas et al. 2002)

Furthermore, it is proposed that a short 20-residue peptide derived from the third ankyrin repeat with the same sequence as fragment 84–103 of p16 can be selected for its ability to bind and inhibit Cdk4 *in vitro* (Fahraeus et al. 1996). Molecular dynamic simulation studies have revealed that this area covers a large number of residues involved in the relevant interactions including some residues related to Cdk4 selectivity (Glu^88^, Gly^89^ and Asp^92^); Phe^90^ that is involved in the distortion of the ATP-binding site and Asp^84^ that is important for binding to Cdk4 (Villacanas et al. 2002). According to docking experiments using Global Range Molecular Matching (GRAMM) calculations, the overall surface contact is primarily between loops 1 and 2 of p16. However, in this study, the first two β strands of 58 N-terminal amino acids of Cdk4, which was modeled based on Cdk2, was used as receptor in the docking calculations (Byeon et al. 1998).

Mutations of loop3 residues led to little change in the inhibitory activity of p16 activity (Yuan et al. 2000).

Since ankyrin repeats are important motifs in protein-protein interactions, it is quite possible that ankyrin repeat structures of p16 are directly involved in the binding to Cdk4. Here to evaluate the interaction tendency and binding affinity of different domains of p16, we have created eight p16 truncated forms, containing various loops and ankyrin motifs by homology modeling and assessed the interaction with Cdk4 via docking protocols.

The energy scores obtained from this study has allowed us to compare these truncated forms and rank them according to their affinity for Cdk4. This experiment can lead us toward the better and faster screening of these truncated forms and reduce the cost of further experimental analysis.

## Materials and Methods

### Tertiary structure determination

Based on the previous studies and considering the most critical regions and amino acids of the tumor suppressor p16^INK4a^ for interaction and inhibition of Cdk4, eight truncated structures were created.

The tertiary structures were determined by homology modeling using the 3D structure of p16, available from the Protein Data Bank (entry 1A5E) as template by ProSAL (Protein Sequence Analysis Launcher), GENO3D web server (http://geno3d-pbil.ibcp.fr/cgi-bin/d3_geno3d2.p1). Due to the truncation, geometry optimization of the obtained structures was carried out with Swiss-PdbViewer 3.7 (2001). The validity of the modeled structures was assessed using the program PROCHEK provided by the GENO3D full model analysis.

### *In Silico* interaction studies

Docked conformations and interaction energies were obtained using the protein-protein docking program HEX 4.5 (2005) and DOT 1.0 Beta, ClusPro server (Comeau et al. 2004a; Comeau et al. 2004b).

During dock operation by HEX using the Macro Docking option, the free energies were calculated based on shape complementarity only and shape/electrostatics as types of correlation using a default grid spacing of 0.6 Å and full rotation of the ligand and the receptor about their own centroids. The program retains a summary of the 10,000 highest scoring orientations, of which the best 500 orientations were retained for viewing.

Docking with DOT was done using a 1 Å grid spacing considering surface complementarity function to sample approximately 10^10^ putative conformations, of which the top scoring 20,000 were retained for empirical free energy filtering and clustering by desolvation and electrostatics. The default values of 1,500 structures with the lowest electrostatic energy and 500 structures with the lowest desolvation free energy were retained and the clustering radius of 7 Å was considered to give more appropriate results.

In all cases, the entire molecular surfaces were utilized in the docking, with no consideration of the active site. The average computational time used for a complex was approximately 6 h for HEX and 4 h for DOT per complex. Hex was performed on a IBM compatible computer running at 1 GB RAM and 2.8 GHz Dual Xeon CPU.

## Results

### Generation of different 3D truncated structures

To identify the most critical regions of p16 involved in the interaction with Cdk4, we generated eight different truncated structures (Table 1) namely A (containing ANKII, III and loops 1,2,3), B (containing ANKI, II and loop 1, 2), C (containing ANKI and loop 1), D (containing ANKII, III and loop 1,2), E (containing ANKIII, and loop 2,3), F (containing ANKII, III, IV and loop 2,3), G (containing ANKIII, IV and loop 2, 3) and H (containing ANKIII, IV and loop 3).

**Figure 3:**
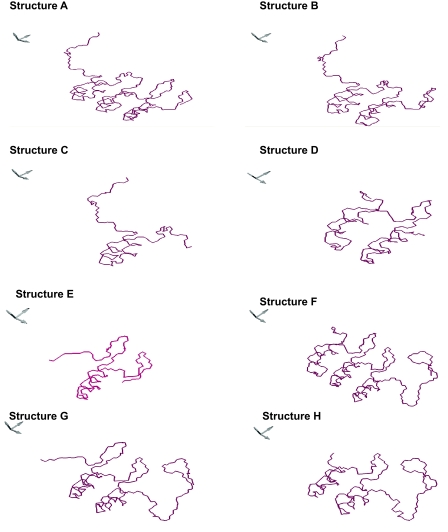
Different 3D truncated structures generated by homology modeling. **A**) 44 amino acids deletion of C-terminal; **B**) 76 amino acids deletion of C-terminal; **C**) 47 amino acids of N-terminal; **D**) amino acids 42–103; **E**) amino acids 66–114; **F**) 41 amino acids deletion of N-terminal **G**) 65 amino acids deletion of N-terminal **H**) 79 amino acids deletion of N-terminal.

Structures were generated by homology modeling. Three models were generated in pdb format in each case, of which the final model was selected considering the lowest free energy (kcal/mol). The Ramachandran plots provided by the GENO3D full model analysis reported that 98.9%, 98.4%, 94.6%, 98%, 100%, 97.9%, 100%, 98.4% of the residues fell within the allowed regions according to truncated structures: A, B, C, D, E, F, G and H respectively. Figure 3 shows the tertiary structures generated by homology modeling.

### Interaction analysis

#### HEX results

**Figure 4:**
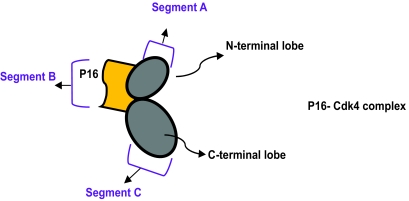
Cdk4 cartoon representation p16-Cdk4 complex and the N and C lobes. Segments A, B and C are represented. The deep cleft between the upper and lower lobe harbors the ATP binding site and the catalytic domain. p16 binding induces allosteric changes by rotating the two lobes thus distorting the ATP binding site (Ortega et al. 2002).

Table 2 lists the docking results of p16-truncated structures and Cdk4, performed by HEX. Both the lowest interaction free energy and the site of interaction are shown. Cdk4 consists of an N-terminal lobe, a C-terminal lobe and a deep cleft at the junction of the two lobes that harbors the ATP binding site and the catalytic domain. p16 binds to the two lobes of Cdk4, predominantly the N-terminal lobe (Coleman et al. 1997; Russo et al. 1998); which we defined as segment B in our analysis. We considered the tip of N-terminal lobe as segment A and the end of C-terminal lobe as segment C for easier reference (Fig. 4).

Considering the shape complementarity alone, it can be deduced that p16 wild type (containing the four ankyrin repeats) shows the best interaction tendency with the lowest free energy of the p16-Cdk4 complex. Truncated forms B, F and H are the next best structures according to the calculated free energy and the free energies were comparable to that of p16 wild type. The truncated forms are bound to Cdk4 with tendency to the segment at the junction of the two lobes (the non-catalytic side) in the same way as found in p16.

Although structure A presents the next best free total energy of the complex, the interaction site of Cdk4 i.e. segment C is not the true binding site. G and C structural complexes with Cdk4 have shown the lower ranking total free energy and D and E complexes show the worst free energy among the truncated forms.

The ranking pattern data considering both shape and electrostatics are somehow different. According to these data, structure G presents the best interaction tendency into the Cdk4 cleft according to the total free energy (Table 2). p16 wild type is the next best fit structure with a close quantity of total energy. The total energy obtained for structures F and H are almost the same and similar to the data pertaining to the shape only correlation. Structure A is the next best fit complex with Cdk4, similar in ranking as is seen in the shape only data.

Regarding structure B, there is a great difference in the interaction energy results obtained from electrostatic factors in comparison to the shape only measurements. The data based on the shape only indicate second best free energy, while considering electrostatic factors, this structure is in moderate range. Finally, the structures C, D and E are ranked in the last positions with the lowest absolute scores of total free energy and/or the interaction tendency to the improper region of Cdk4 (Table 2).

#### DOT results

Docking results achieved from DOT server are presented in Table 2. Similar to the HEX shape only data, p16 wild type shows the best interaction tendency. Allowing for some variations with structure D, truncated forms H, G and F have got nearly the same total free energy. However, there is a little difference in the ranking of some of the structures like structure A and D based on the total energy score in comparison to the data obtained from HEX shape only and shape and electrostatics correlation. The DOT data indicates that similar to the HEX data, structure E and C are among the worst complex structures considering the total free energies.

## Discussion

Docking is a computational process used for finding the best matching between two molecules and has been heavily applied in the rational drug design. Protein-protein docking, owing to the sizes of the molecules is the most challenging task facing the proteomics era (Halperin et al. 2002). Most of the structures in the Protein Data Bank (PDB) are single proteins and as structural genomics efforts increase the rate of determining monomeric structures, the fraction of complexes in PDB will decrease (Gray, 2006).

Accurate prediction of results from docking methods could therefore provide substantial structural knowledge of protein complexes, from which functional information can be inferred or experiments can be designed (Smith and Sternberg, 2002).

In this regard, HEX, based on the new method introduced by Ritchie and Kemp applies spherical polar Fourier correlations to accelerate the docking and superposition calculations, 10 to 100 times faster than conventional FFT docking algorithms (Ritchie and Kemp, 2000).

Shape complementarity is an essential criterion with powerful scoring function in bound and unbound protein-protein docking, where the structures have been determined separately (Norel et al. 1999; Norel et al. 2001). Spherical polar Fourier correlation has allowed us to consider not only shape complementarity but also increase the contribution of the electrostatic correlation in our docking analysis by using the shape and electrostatic calculation option. HEX has performed well in the CAPRI blind protein-protein docking trials (Mendez et al. 2005; Mendez et al. 2003). Based on the previous studies, there is essentially no limit to the size of the proteins which may be docked with this approach (Ritchie, 2003).

DOT ClusPro algorithm, another rigid body docking program, is automated and based on the Fast-Fourier Transform correlation technique. During DOT docking, only the shape complementarity is used and in the next step, the top scoring putative conformations are filtered by desolvation and electrostatics calculations. The structures then clustered using a hierarchical pairwise RMSD algorithm; this algorithm then selects the centers of the most populated clusters as predictions of the unknown complex. A limitation of the server is the size of the proteins; no more than 11,999 atoms for the receptor and no more than 4,700 atoms for the ligand after minimization are allowed (Comeau et al. 2004a; Comeau et al. 2004b). The implementing algorithm in ClusPro has been successfully tested in blind CAPRI experiment where it has generated some of the best predictions for the given target structures (Camacho and Gatchell, 2003).

Here, we have modeled eight truncated p16 forms with different combinations of ankyrin motifs via homology modeling. After energy minimization and checking the Ramachandran plot of each form with PROCHECK, available at the GENO3D full model analysis, we tested the complex docking modes with Cdk4 as the receptor to compare the interaction tendencies of a special motif or a group of them within p16.

Table 2 presents the lowest free energy of binding using two docking programs HEX 4.5 and DOT 1.0 Beta. Based on the previous knowledge from HEX, we have utilized N = 30 as steric scan correlation that improves the average rank of good docking orientations compared to the softer N = 25.

**Figure 5:**
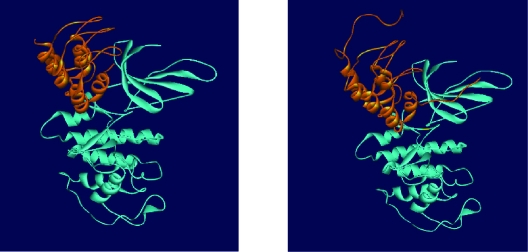
**a**) Complex structure of p16-Cdk6 available at Protein Data Bank (entry 1BI7). **b**) p16-Cdk6 complex obtained in our study from HEX docking program. p16 has interacted with Cdk6 in the similar pose and location as indicated in the crystal structure.

We have got considerable binding modes and free total interaction energies during unbound docking of the eight p16 truncated forms and Cdk4. In order to further validate our data, we have performed docking on p16 eight truncated structures and Cdk6. The 3D structure of p16-Cdk6 complex is available and the binding mode has been determined through previous studies (Yuan et al. 2000). The binding site of p16 and the truncated structures obtained from HEX docking program have been the same as p16-Cdk6 complex and the ranking of free interaction energies are comparable with our data. Figure 5 represents the available p16-Cdk6 complex and the docked complex formed using HEX.

Our results indicate that using N = 30 as steric scan correlation together with considering combined shape and electrostatics provides a better average rank compared to shape-only correlations using HEX. Since most of the p16 residues involved in Cdk4 binding are charged amino acids it could be deducted that electrostatic interactions between charged residues contribute significantly to the interaction between p16 and Cdk4 (Byeon et al. 1998). Interestingly, a similar behavior is observed in ranking total free energies obtained from DOT and HEX shape and electrostatic consideration.

In this regard, ankyrin repeat III which contains the least number of hydrophobic residues among the four ANK repeats may have the most important role in providing a good total interaction energy toward Cdk4. Based on our results from DOT and HEX/shape and electrostatics, in structure G, which contains ANK repeats III and IV together with the intervening loops 2 and 3, the interaction energy is comparable to that of p16 wild type. Structure G even demonstrates a lower total free energy compared to structure F, which contains additional regions of ankyrin repeat II and loop 1. Comparing structures G and H, the docking results suggest that addition of loop 2 may increase the interaction tendency toward Cdk4. This is in parallel with the previous studies that indicate the importance of β-hairpin loop connecting the second and third ankyrin repeats for Cdk4/6 binding (Zhang and Peng, 2000). Considering the results obtained from docking of structure F, one can conclude that the existence of ANK I together with loop 1 and the N-terminal segment, as is seen in structure C, is not critical for Cdk4 interaction. Structure C does not show a proper interaction tendency toward Cdk4 and is among the worst structures in ranking the truncated structures according to the results obtained from both HEX and DOT docking programs. As indicated here, our analysis suggests that loop 1 may not act alone in Cdk4 interaction as discussed above; while loop 2 could be more important, the combination of loops 2 and 3 provide a better interaction.

**Figure 6:**
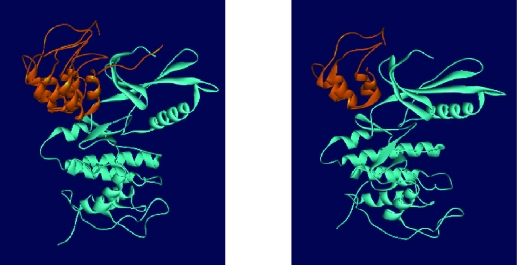
Representation of complex molecules docked by HEX. **a**) p16 binds to both the N and C-terminal lobes of Cdk4, predominantly the N-terminal lobe. **b**) truncated structure H interacts with Cdk4 in almost the same manner as the full length molecule.

The ankyrin motifs possess highest primary sequence conservation in the ankyrin motifs and mutations in these regions seem to disrupt the function of the protein. While few natural mutations can be detected in the flexible N- and C-terminal segments, in most of the mutants, at least one change in the conserved ankyrin sequence has occurred. It was shown that point mutations in the conserved ankyrin sequence affected the activity, and mutation in other regions had no apparent effect. (Byeon et al. 1998; Yuan et al. 1999; Yang et al. 1996). The comparison between total free energy of structure H and F may demonstrate that ankyrin repeats III and IV with intervening loops 2 and 3 can cover for Cdk4 interaction and ankyrin IV motif may play somewhat a helper role in the interaction since its removal has weakened the obtained free energy of binding and also changed the binding site on Cdk4 in structure A. It is previously showed that the deletion of the fourth ankyrin repeat abolished the activity completely (Yang et al. 1995). Figure 6 presents the complex of p16-Cdk4 and truncated structure H-Cdk4, as a sample with good interaction profile, after being docked with Hex.

Our data indicates that ANK repeat III with the adjacent loops 2 and 3 alone in structure E has not represented a good docked model with Cdk4 despite the previous claim that a 20-residue synthetic peptide corresponding to amino acids 84–103 of p16 interacts with Cdk4 and inhibits the phosphorylation of pRb *in vitro* (Fahraeus et al. 1996). We have performed docking on this 20-residue fragment of p16, however the obtained energy score, (−521.5 KJ/mol from HEX shape only considerations) is not comparable to that of p16 wild type and other truncated forms. On the other hand, the shape/electrostatics total energy was −1184.2 KJ/mol and more reasonable. The reason for this observation could be attributed to the small structure of the 20-residue peptide and a special conformation it adopts in solution upon binding to Cdk4 and therefore blockage of the catalytic center of the kinase (Zhang and Peng, 2000). However, in our applied rigid docking programs, such conformational changes were not allowed within the components upon complex formation. In addition, it has been reported that a structure containing a single ANK III or IV are predominantly unstructured in solution and cannot fold independently (Zhang and Peng, 2000).

Addition of ANK II to the structure E, as seen in structure D, causes an increase in interaction tendency toward Cdk4 based on DOT docking results. According to the X-ray data from p16-Cdk6 complex, loops 1 and 2 are more important in Cdk6 binding than loop 3 (Yuan et al. 2000). However, in our docking analysis addition of loop 3 together with ANK IV increases the interaction tendency toward Cdk4 in structure F to a large extent in comparison with the lowest free energy of structure D which lacks loop 3 and ANK IV. As discussed above, deletion of ANK I and loop 1 does not have important effect on Cdk4 interaction in structure F in comparison to the p16 full length total free energy according to the two applied docking programs.

When we consider p16 structure into two halves, the N-terminal half consisting of ankyrin repeats I and II plus loops 1 and 2 (structure B in our study) and the C-terminal half including ANK III and IV together with loop 3 (structure H): comparing the total interaction energy of these two structures achieved from DOT and HEX shape and electrostatics, one can propose that ANK III and IV motifs together with the intervening loop 3 may represent a more important region in interaction with Cdk4 than ANK I and II plus loop 1 and 2. This assumption is consistent with the previous studies indicating mutations are present with high frequency in three regions: residues 71–76, 80–102 and 107–127 that lies in loop 2, entire ankyrin repeat III, and from loop 3 to the beginning of helix IVB (Byeon et al. 1998). The residues involved in the selectivity, distortion of the ATP-binding site and binding toward Cdk4 span through ankyrin III segment (Villacanas et al. 2002). Moreover, it has been previously proposed that ANK II and III appear to be more critical to p16 function and mutations in ankyrin repeats I and IV are less likely to disrupt p16 function (Yarbrough et al. 1999). Taking all into account, our analyses reveal that ankyrin repeat III may play the most critical role for Cdk4 binding, however this feature is just true if the other segments like loop 2, 3 and ANK IV exist. This assumption will be analyzed experimentally by our team in the near future.

The limitations of the recent study, include complex and unexpected molecular interactions. p16 and other truncated forms can be docked without serious considerations for the induced conformational changes upon docking, as it can be seen that almost all of them were located correctly in the actual binding site of Cdk4 with comparable energy scores. Predicted complexes can be used to direct experimental studies as it is now being designed and considered to be done in our research group, which can confirm the data obtained from docking analysis.

Considering all of the above, it can be deduced that structures containing ANK III and IV with adjacent loops are suitable candidates for inhibition of Cdk4. Besides the conserved ANK motifs are important for Cdk4 interaction and the intervening loops are critical in a way that removal of them, for instance loop 2, can weaken the interaction toward Cdk4.

## Figures and Tables

**Table 1:** Specifications of ANKs motifs and loops of different truncated structures studied.

Truncated structure	Ankyrin motifs and loops								
**Full length**	N	ANK I	L 1	ANK II	L 2	ANK III	L 3	ANK IV	C
**A**	N	ANK I	L 1	ANK II	L 2	ANK III	L 3		
**B**	N	ANK I	L 1	ANK II	L 2				
**C**	N	ANK I	L 1						
**D**			L 1	ANK II	L 2	ANK III			
**E**					L 2	ANK III	L 3		
**F**			L 1	ANK II	L 2	ANK III	L 3	ANK IV	C
**G**					L 2	ANK III	L 3	ANK IV	C
**H**						ANK III	L 3	ANK IV	C

**Table 2:** HEX and DOT docking results, according to E-total (KJ/mol) and interacting segment (pose). The binding sites are assumed to be unknown and the entire molecular surfaces are considered.

Target	ANKs and terminals	Loops	HEX E-total (shape only)	Pose	HEX E-total (shape and electrostatic)	Pose	DOT E-total (shape only)	Pose
**Full length**	N terminal I, II, III, IV C terminal	1, 2, 3, 4	−732.7	B	−19,905.6	B	−24.46	B
**A (δ1–112)**	N terminal, I, II, III	1, 2, 3	−675.2	C	−9,435.1	C	−1.95	B
**B (δ1–80)**	N terminal, I, II	1, 2	−719.4	B	−8,925.1	B	−11.83	B
**C (δ1–47)**	N terminal, I	1	−654.3	B	−5,227.2	B	0.45	B
**D (δ42–103)**	II, III	2, partly 1 & 3	−630.2	B	−1,390.9	A	−16.81	C
**E (δ66–114)**	III	2, 3	−603.8	B	−1,034.6	B	−3.83	B
**F (δ42–156)**	II, III, IV, C terminal	2, 3, partly 1	−705.6	B	−15,569.2	B	−17.27	B
**G (δ66–156)**	III, IV, C	2, 3	−662.1	B	−22,079.4	B	−19.48	B
**H (δ80–156)**	III, IV, C terminal	3	−698.8	B	−15,867.6	B	−17.38	B
